# MicroRNAs signatures, bioinformatics analysis of miRNAs, miRNA mimics and antagonists, and miRNA therapeutics in osteosarcoma

**DOI:** 10.1186/s12935-020-01342-4

**Published:** 2020-06-17

**Authors:** Babak Otoukesh, Mehdi Abbasi, Habib-o-Lah Gorgani, Hossein Farahini, Mehdi Moghtadaei, Bahram Boddouhi, Peyman Kaghazian, Shayan Hosseinzadeh, Atefe Alaee

**Affiliations:** 1 Orthopedic Surgery Fellowship in Département Hospitalo-Universitaire MAMUTH « Maladies musculo-squelettiques et innovations thérapeutiques » , Université Pierre et Marie-Curie, Sorbonne Université, Paris, France; 2 Department of Orthopedic Surgery, Bone and Joint Reconstruction Research Center, Iran University of Medical Science, Postal code : 1445613131 Tehran, Iran; 3 Brain Mapping Research Center, Shahid Beheshti University of Medical Sciences, Tehran, Iran; 4 Department of Orthopedic and Traumatology, Universitätsklinikum Bonn, Bonn, Germany; 5 Department of Orthopedic Surgery, Boston Children’s Hospital, Harvard Medical School, Boston, MA USA; 6 Department of Information Sciences, Tehran University of Medical Sciences, Tehran, Iran

**Keywords:** MicroRNAs, Osteosarcoma, Targets, Therapy

## Abstract

MicroRNAs (miRNAs) involved in key signaling pathways and aggressive phenotypes of osteosarcoma (OS) was discussed, including PI3K/AKT/MTOR, MTOR AND RAF-1 signaling, tumor suppressor P53- linked miRNAs, NOTCH- related miRNAs, miRNA -15/16 cluster, apoptosis related miRNAs, invasion-metastasis-related miRNAs, and 14Q32-associated miRNAs cluster. Herrin, we discussed insights into the targeted therapies including miRNAs (i.e., tumor-suppressive miRNAs and oncomiRNAs). Using bioinformatics tools, the interaction network of all OS-associated miRNAs and their targets was also depicted.

## Introduction

### MicroRNA and cancer

MicroRNAs (miRNAs) are considered as a class of non-coding RNAs, which their expression patterns are demonstrated to be tissue and cancer-type specific [1]. MiRNAs are not only detectable in cells but also in various bio-fluids such as plasma and serum, as well as in follicular fluid, etc., namely extracellular miRNAs (ECmiRNAs) [2–4]. Circulating miRNAs from tumor cells have attracted the attention of researchers because of their diagnostic and prognostic potential, when are capable of preventing a novel opportunity for early prediction of cancer and treatment. It is noteworthy that miRNAs are capable of regulating their target gene by either induction of miRNA degradation or abrogation of miRNA translation [5–7]. Aberrant expression levels of miRNAs have been found to be associated with the initiation and progression of many kinds of cancers in tissues and cell lines, such as osteosarcoma (OS) [8, 9].

MiRNAs are capable of regulating 90% of protein-coding genes [10]. Mature miRNAs often play an important role in the pathogenesis of OS as an oncogenic or tumor suppressor agent because changes in miRNA regulation seem to be markedly associated to cell proliferation, adhesion, invasion, migration and metastasis, as well as apoptosis [11, 12]. Consequently, these molecules may be regarded as good strategies for the development of prognostic markers of various malignancies.

It is noteworthy that a given miRNA may have different miRNA targets; on the other hand, it should be taken into account that multiple miRNAs are capable of regulating a given miRNA target. Nevertheless, the interplay between miRNAs and targeted genes is complex, when the intricate interplay is not obviously revealed [13].

The better in-depth understanding of the molecular mechanisms of miRNAs using pathway-based exploratory evaluations, mapping and characterization of miRNA can pave not only the way to characterize the pathogenesis of OS, but also provide miRNA-based therapy for improving the prognosis of OS patients [2, 5, 7, 13]. Furthermore, functional assessment of single miRNA can be great of importance to determine its role in the pathogenesis and tumorigenesis of OS [2, 7, 13]. Therefore, miRNAs are undergoing clinical evaluation for many types of malignancies (Tables 1 and 2).

**Table 1:** Clinical trial development by MiRNA; data were adapted from https://clinicaltrials.gov

Study title	Conditions	Interventions	Study type and phase
Plasma microRNA, lung cancer	Lung cancer	Screening	Interventional study, not applicable
MicroRNA-155 and telomerase reverse transcriptase, non-muscle invasive bladder cancer	bladder cancer and disease	Diagnostic method: miRNA -155 Diagnostic method: Human telomerase reverse transcriptase	interventional, not applicable
Plasma microRNAs, pelvic gynecologic tumors	Ovarian cancer Endometrial cancer	Other: blood sample	Interventional study, not applicable
Circulating miRNAs, breast cancer	Breast cancer	Drug: tamoxifen, letrozole, anastrozole, exemestane	Interventional study, Phase 4
A 6 microRNA Tool for Stratifying Stage II colon cancer	Colonic Chemotherapy	A 6 microRNA stratified tool	Interventional study, Not applicable
MicroRNA in NAF, serum, and tissue, breast cancer	Breast Cancer Ductal carcinoma in situ	Drug application: intranasal oxytocin	Interventional study, Phase 2
A multicenter phase I study of MRX34, MicroRNA miR-RX34 liposomal injection	Primary liver cancer SCLC Lymphoma	Drug application: MRX34	Interventional study, Phase 1
MicroRNA involved in cutaneous squamous cell carcinoma	Cancer of the Skin	Genetic: arm A	Interventional study, not applicable
Plasma miRNAs for Predicting Radiosensitivity in Advanced Non-small Cell Lung Cancer	Advanced non-small cell lung cancer	Radiotherapy	Interventional study, not applicable

**Table 2:** Observational clinical trial development by MiRNAs; all data was adapted and collected from https://clinicaltrials.gov

Study title (reference)	Conditions	Interventions	Type
Circulating microRNA for cardiotoxicity in breast cancer	Breast cancer	–	Observational
MicroRNAs Tool for stratifying stage II colon cancer	Colon cancer, effects of chemotherapy	Device: miRNA tool	Observational
MicroRNA processing enzymes dicer and drosha	Skin cancer	–	Observational
MicroRNA blood test for lung cancer screening	Lung cancer	–	Observational
Micro RNAs for prediction of response to androgen deprivation therapy	Prostate cancer	Drug: bicalutamide, leuprolide, goserelin	Observational
Investigating the role of novel molecular profiles, microRNA’s, and their targets in colorectal cancer progression	Colorectal cancer	Biomarker study	Observational
MIRNA profiling of breast cancer	Breast cancer	–	Observational
Circulating microRNA as a tool for primary brain tumors	Brain tumors	–	Observational
The utility of circulating tumour cells and plasma microRNA	Esophageal cancer	Blood draw	Observational
Anti-IMP3 Autoantibody and MicroRNA signature blood tests for detection of metastatic kidney cancer	Kidney cancer	Genetic: gene and protein expression analysis Diagnostic tools: laboratory biomarker	Observational
MicroRNAs for diagnosis of pulmonary cancer	Pulmonary cancer	Blood punction	Observational
Circulating miRNAs. ICORG 10–11, V2	Breast ccancer, Recurrent breast cancer	–	Observational
Lipidomics, proteomics, micro RNAs and volatile organic compounds	Pancreatic neoplasms	blood and bile	Observational
Chemoresistance in epithelial ovarian cancer	Ovarian Cancer	–	Observational
MicroRNA-10b in patients with gliomas	Astrocytoma, oligodendroglioma, oligoastrocytoma	–	Observational
MicroRNAs in neurofibromatosis type 1	Glioma Neurofibromatosis Type 1	–	Observational
Microarray analysis in basal cell carcinoma	Basal cell carcinoma	–	Observational
Microarray analysis of microRNA in cutaneous squamous cell carcinoma	Cutaneous squamous cell carcinoma	–	Observational
MicroRNA expression in renal cell carcinoma	Renal cell carcinoma	–	Observational
The role of microRNA in cutaneous melanoma	Melanoma	–	Observational

MiRNA-targeted therapies have been suggested to be a more promising approach to hamper aggressive biological behavior of OS [14, 15]. Unlike multitude other kinds of cancer, there are no traditional markers found for OS. Therefore, the recognition of novel diagnostic miRNA biomarkers could finally have a prognosis or therapeutic value in this disease [5]; however, metastatic nature of the disease and the histological response after adjuvant chemotherapy is confirmed as the only predictor of event-free survival [5, 16]. As mentioned in Tables 1 and 2, clinical trials are performed to provide novel predictor and markers of response to therapy by evaluating the miRNAs expression patterns in the blood, body fluids, and tissue.

### MicroRNA analysis

Most investigations have used methods such as quantitative real time-PCR, gene arrays and miRNA sequencing for evaluating miRNA profiles at low cost with a high efficiency [17–19]. The conventional methods, including cloning, microarray, and in situ hybridization are considered to be cost consuming techniques [20–23]. All pre-analytical and analytical approaches should be standardized for avoiding higher repercussions of technical biases on miRNA results, therefore, validation of results is needed before the translation of circulating miRNA patterns into a clinical evaluation [17, 24]. By development of bioinformatics, different bioinformatics tools have been provided for managing miRNA biology data and investigating questions [25]

Annotation tools are applied to investigate miRNA biology. A platform for miRNA data should be taken into consideration in this regard. Many tools are developed in the field of annotation associated miRNA tools such as miRBase, Rfam, mirtronPred and MetaMirClust [25–31]. For instance, miRBase is introduced as a searchable database (http://www.mirbase.org) that published 24,521 miRNA loci from 206 species (e.g., 1872 miRNA precursors of human, producing 2578 mature miRNAs) [26].

Structure tools are developed for the prediction and comparison of RNA structure [25, 32]. Structural features of a given miRNA molecule can be elucidated by tools such as ViennaRNA software package [25, 32, 33].

Furthermore, identification tools are widely used based upon next-generation sequencing (NGS) information via various algorithms and tools such as miRDeep (miRDeep/miRDeep2) and miRanalyzer [25, 34, 35] by which miRNA characteristics such as sequence conservation, structural properties (i.e., hairpin and minimum free energy) can be obtained.Moreover, miRNAFold as a fast ab initio method is used for predicting miRNA in the genome [36, 37]. Computational algorithms have provided harmonize experimental strategies for discovering and validating novel miRNAs [23, 37]. Furthermore, network analysis is taken into consideration for providing drug target, as well as for planning novel therapeutic and diagnostic approaches. Network biology is developed to inspect components for deducing valuable data from large transcriptomic datasets, by which metabolic networks depend on each other are capable of showing the behavior of the network biology [38–40].

### Circulating MicroRNAs as key regulator in OS pathobiology

Different clinical studies in the last several years have demonstrated that miRNAs, especially circulating miRNAs in serum are involved in OS development and progression. Therefore, they can potentially be applied as diagnostic and prognostic markers [41].

**Fig. 1:**
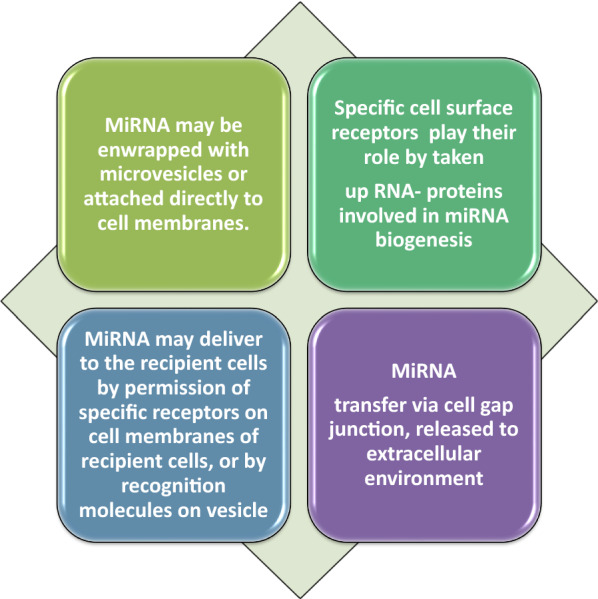
Possible ways to release circulating miRNAs; 3 pathways including extracellular vesicles, RNA-binding proteins and/or transfer through cell gap junction are involved. In addition, the topology of the network was analyzed based on degree metrics in order to find the most important nodes. According to the findings, 5 nodes with large degrees in our large network (i.e., > 9, see green nodes) including BCL2, VEGFA, CCND1, PTEN and MET were identified as potential hub nodes, (Additional file 1, and Fig. 2)

Mature miRNAs are first detectable in serum and plasma, and can thereafter be detected in biological fluids [42–45]. It has been suggested that circulating miRNAs are more likely to undergo selective packaging and release, and their secretion in cells could be linked to a given pathological condition [43, 45, 46],

Ample evidence indicates that the uptake of circulating miRNAs by their target cells is absolutely essential for eliciting their regulatory functions [45, 47]. Pathways involved in the uptake of circulating miRNAs are presented in Fig. 1.

Overall, increasing evidence reveals that the release of ECmiRNAs in the extracellular harsh environment can be mediated for intercellular connection by microvesicles, exosomes, apoptotic bodies, and high density lipoprotein (HDL), as well as argonaute (AGO) protein complex [47]. Therefore, the release of circulating miRNAs from cancer cells play a substantial role in tumorigenesis of recipient cells (normal cells). It is noteworthy that further clarification of these pathways will require an in-depth understanding of the mechanisms that underlie release of cellular miRNAs, regulation and uptake of circulating miRNAs in order to elucidate cancer biology. An increasing body of evidence suggested that ECmiRNAs can be involved in the pathophysiological condition of cancer. Therefore, these ECmiRNAs may be delivered to the recipient cells through many pathways, by which they are capable of regulating translation of their target genes. The evaluation of single ECmiRNAs (e.g., exosomal miRNAs or protein-bound miRNAs) may be useful in comparison with total ECmiRNAs. As a matter of fact, ECmiRNA investigations need technological advancement with standardized protocols for obtaining reliable findings in terms of disease biology, where may result in the development of new therapeutic strategies [47].

Microvesicles may be secreted by many cell types including many types of cancerous cells, B cells, endothelial cells, dendritic cells and neurons [48–50]. Circulating miRNAs are considered as noninvasive biomarkers, where a variety of strategies are being conducted in clinical studies for determining values of circulating miRNAs. Circulating miRNAs have been introduced as a potential marker for early diagnosis and monitoring of OS. Therefore, validation of these markers in clinical trials is currently needed. Most investigations have used methods for evaluating circulating miRNAs such as quantitative real time-PCR, gene arrays and sequencing [51]. Circulating miRNAs are summarized in Table 3 for monitoring of OS, where a list of miRNAs are provided based on their oncogenic and tumor suppressor activity consisting of clinicopathologica status.

**Table 3:** Circulating miRNA(s) in patients suffering from osteosarcoma

OncomiRNA and tumour suppressor miRNA(s)	Clinical findings	References
miR-9^Om^	Increased levels of miR-9 were found to be related to higher TNM stage, distant metastasis and large tumour size; as well as poor S	Fei et al. [233]
miR-17^Om^	Increased level was detected in OS patients, where it was linked to poor S; Serum miR-17 levels was reported to be linked to tensin homolog (PTEN) expression and tissue phosphatase	Li et al. [234]
miR-24^Om^	Increased serum and tissue miR-24 levels were detected in OS patients	Sun et al. [235]
miR-27a^Om^	Higher miR-27a levels was detected to be linked to higher clinical stage, and distant metastasis; Higher miR-27a levels was found to be correlated with poor response to chemotherapy, and was capable of differentiating OS from HC; it serves as an independent prognostic marker of unfavourable survival	Tang et al. [236]
miR-34b^T^	Decreased plasma level and low tissue expression of miR-34b were detected, lower level has been found in metastatic patients, it was considered as circulating tumour suppressor miRNA	Tian et al. [58]
miR-25-3p^Om^	miR-25-3p level was increased in OS patients; its increase was linked to poor PFS, and it was capable of differentiating OS from healthy control.	Fujiwara et al. [63]
miR-29 family^Om^	Higher miR-29a/b/c levels were detected to be associated with OS in evaluated patients; this markers serves as independent prognostic factors of unfavourable survival	Hong et al. [237]
miR-21, miR-143, miR-199a-3p^Om^	MiR-21 levels increased in OS, whereas miR-143 and miR-199a-3p levels were decreased in OS Decreased levels of MiR-21 and miR-143 were found to be linked to metastasis and histological subtype; Low level of miR-199-3p was linked to histological subtype Increased miR-21 was found in the blood, where its high expression was linked to higher Enneking stage and chemotherapeutic resistance	Ouyang et al. [59]; Yuan et al. [60]
miR-95-3p^Ts^	Decreased serum level of miR-95-3p was indicated to be linked to clinical stage, metastasis and chemotherapy response. It was considered as circulating tumour suppressor miRNA	Niu et al. [238]
miR-125b^Om^	Decreased level of miR-125b was linked to advanced tumour stages Furthermore, it was capable of differentiating chemotherapy-resistant patients from chemotherapy-sensitive	Luo et al. [53]
miR-133b miR-206^Ts^	MiR-133b and miR-206 downregulation were found to be related to advanced tumour grade, metastasis and recurrence in OS patients’ sera, as well as poor response to chemotherapy in patients. Decreased levels of both miRNAs is attributed to 18 months’ survival time, which is indicated to be a shorter survival in comparison with the mean 24 months survival time in patients with decreased level of only one miRNA; miR-133b and miR-206 may present opportunity as non-invasive biomarker for diagnosis and prognosis of OS	Zhang et al. [239]
miR-148a^Om^	Increased expression of circulating miR-148a was linked to increased tumour size and distant metastasis and a negative association with five-year survival in OS patients, where it was revealed to be an independent prognostic factor of unfavourable survival; As a matter of fact, 148a has been suggested to be a vindicator marker for progressive phenotype, and a novel diagnostic biomarker in the peripheral blood for determining poor prognosis in patients suffering from OS	Ma et al. [240]
miR-152^Ts^	Lower serum and tissue levels of miR-152 levels were linked to Enneking and metastasis in OS patients; decreased level revealed to be capable of differentiating OS from HC, and serves as an independent prognostic factor for unfavourable survival	Wang et al. [241]
miR-196a, miR-196b^Om^	Increased levels of tissue and serum miR-196a and miR-196b were detected; Higher serum miR-196a and miR-196b and their co-expressions were linked to advanced tumour grade, recurrence and metastasis status in OS patients; expression levels of both MiRs were related to unfavourable survival	Zhang et al. [242]
miR-195-5p, miR-199a-3p, miR-320a, miR-374a-5p^Om^	MiRs levels were elevated in OS patients and their downregulation were found in postoperative samples; MiR-195-5p and miR-199a-3p were found to be linked to metastasis status, whereas miR-199a-3p and miR-320a levels were related to histological subtype	Lian et al. [62]
miR-199a-5p^Om^	Increased levels of miR-199a-5p levels were detected in OS patients; its decreased level were found in postoperative samples; MiR-199-5p was capable of differentiating OS from healthy control	Zhou et al. [243]
miR-221^Om^	Increased level of miR-221 in OS patients, and its tissue and serum levels was found to be prognostic factor of unfavourable survival; MiR-221 was found to be capable of differentiating OS from HC	Yang et al. [244]
miR-223^Ts^	Decreased level of miR-223 was linked to advanced clinical stage and distant metastasis	Dong et al. [245]
miR-300^Om^	Increased tissue and serum miR-300 levels were detected in OS patients; higher clinical stage and distant metastasis were found to be linked to increased level of miR-300 levels; serum levels found to be reduced in OS patients after curative surgery; serum miR-300 was suggested as an independent prognostic marker of unfavourable survival	Liu et al. [246]
miR-326^Ts^	Lower serum and tissue levels of miR-326 levels were detected in OS patients; it serves as circulating tumour suppressor miRNA; MiR-326 was capable of differentiating OS from HC; Decreased serum miR-326 levels were linked to higher clinical stage and distant metastasis, whereas its decrease tissue level was related to distant metastasis; its lower serum level was suggested to be an independent prognostic factor of unfavourable survival	Cao et al. [247]
miR-497^Ts^	A circulating tumour suppressor miRNA; lower miR-497 levels was found to be liked to response to chemotherapy, and clinical stage, distant metastasis	Pang et al. [248]

*Ts* tumour suppressor miRNA(s), *Om* oncomiRNAs

Other miRNAs that are correlated with the OS development include the following: miR-20a-5p, miR-106a-5p, miR139-5p, miR451a, miR16-5p, miR-25-3p, and miR-425-5p demonstrated to be weakly expressed in the serum of patients suffering from OS in comparison with healthy controls. Aforementioned miRNAs have been suggested to serve as diagnostic markers for differentiating healthy cohort and OS [52].

The decreased expression level of circulating miR-125b has been indicated to be linked to poor disease-free survival in patients suffering from OS, and this miRNA was capable of predicting the cisplatin resistance in patients with OS, where decreased miR-125b was related to high tumor stages [53]. Decreased level of miR-125b as a tumor suppressor has been found in human OS tissues [54, 55], and its weak level was found to be related to higher TNM stage, large tumor size, and metastasis [56, 57].

Plasma miR-34b was introduced as a new potential therapeutic marker for OS, where its expression was causally linked to metastasis, thus leading to development of OS [58]. A three-miRNA signature including down-regulation of plasma levels of both miR-199a-3p and miR-143 and up-regulation of plasma miR-21 level has been demonstrated in patients with OS, which were able to discriminate OS from controls subjects [59]. Yuan et al., reported that higher Enneking stage and chemotherapeutic resistance can be markedly associated with serum miR-21 level, where its serum level can serve as an unfavorable prognostic factor for OS [60].

Lower serum and tissue miR-598 levels have been revealed to be associated with migration, invasion and proliferation of OS cells. A growing body of evidence demonstrates that miR-598 is involved in OS progression by targeting platelet-derived growth factor (PDGF) -β and mesenchymal epithelial transition (MET), as well as modulation of osteoblast differentiation in the microenvironment, indicating its potential as diagnostic, prognostic, and therapeutic marker [61].

Up-regulation of four plasma miRNAs (miR-320a, miR-374a-5p, miR-195-5p, and miR-199a-3p,) have been previously identified in OS patients, of which plasma levels of miR-195-5p and miR-199a-3p have been found to be linked to the metastatic OS, whereas miR-199a-3p and miR-320a plasma expression levels were revealed to be related to histological subtype. Moreover, these miRNAs were capable of discriminating OS patients from healthy subjects. Postoperative up-regulation of these plasma miRNAs was also detected [62].

Circulating miR-25-3p level has been found to be increased in OS in the validation cohort. In addition, serum miR-25-3p levels were revealed to be a predictor of patient prognosis as a blood-based biomarker, where its association with tumor burden has been revealed in both invivo experiment and patients [63]. Emerging evidence suggests that down-regulated serum miR-101 level can be markedly linked to higher clinical stage and distant metastasis, as well as poor overall survival and recurrence free survival, suggesting its potential for OS diagnosis, with a favorable specificity*/*sensitivity [64].

Another study indicated that low serum miR-375 level could be linked to high clinical stages, increased tumor size, and distant metastasis, as well as chemoresistance after surgery in OS. Furthermore, the miR-375 expression may be a novel target for diagnosis, prognosis, and chemosensitivity prediction in OS patients [65]. It is noteworthy that efforts are at the beginning of assessing miRNAs expression patterns in OS initiation and progression.

### PI3K/AKT/MTOR pathway -related miRNAs and MAPK pathways-related MicroRNAs

The tumor suppressor phosphatase and tensin homolog (PTEN) (200 kb gene on hromosome10q23) suffers loss of function in many types of malignancies such as bone metastases, and OS, which is described to act as negative regulator of the PI3K/Akt activation [66], which may be influenced by genetic mutation, loss of heterozygosity (LOH) of chromosomal regions, DNA promoter hypermethylation, and miRNAs-mediated gene expression [5, 67]. PTEN is a multifunctional tumor suppressor that is negatively involved in the regulation of the Akt pathway for preventing cell proliferation [5]. PTEN mRNA level has been previously found to be inversely linked to up-regulation of oncogenic miR-92a, miR-17, miR-130/301 families and miR-26 families. PTEN is involved in antagonizing signaling via the PI3K/PTEN/Akt pathway, which was demonstrated to play a substantial role in progression and development of OS through inducing cell proliferation and inhibiting apoptosis [68].

PTEN as a target of miR-26a, miR-106b-25 cluster (7q22.1) and miR-17-92 cluster family (13q31.2) have been confirmed to be decreased in OS [68–71], where plays a key role in the development of OS by inducing cell proliferation and suppressing apoptosis. In the literature, miR-17-92 cluster, miR-106b-25 paralog cluster and miR-106a-92 clusters have been verified to be increased in OS cell lines and different cancers [68, 72, 73]. Accumulating evidence indicates up-regulation of miR-17-92 in OS, as well as up-regulation of miR-106a (miR-106a-92 cluster) and miR-106b (miR-106b-25 clusters) [74, 75].

MiR-17 was up-regulated in OS tissues by which PTEN could be inhibited via binding to its 3′-UTR, indicating that miR-17 as oncogene has an important role in OS cell growth, migration, and invasion [76].

Increasing evidence suggests that miR-221 plays a substantial role in cancer development. MiR-221 was capable of promoting OS cell proliferation, invasion and migration at least partly via reducing PTEN [77]. Up-regulation of miR-221 was identified to be capable of inducing cisplatin resistance and cell survival in both human OS cell (SOSP-9607) and MG63 partly via PI3K/PTEN/Akt pathway through targeting PTEN pathway, while knockdown of miR-221 has been revealed to be involved in cell growth inhibition, the increase of cisplatin resistance and induction of cell apoptosis [78], showing its potential as a therapeutic strategy for the prevention of OS.

A study suggested that over-expression of miRNA-21 as an oncogene could be able to activate the PTEN/PI3K/AKT signaling via down-regulating the expression level of PTEN in MG-63 as OS cell line, where its expression level was found to be positively linked to the expression of AKT/p-AKT, suggesting that miR-21 is implicated in regulation of the cell proliferation and invasion as shown previously on MG-63 cells [79]. PTEN has been suggested as a target of miR-21, which is capable of activating PI3K/Akt pathway via inhibiting PTEN expression level [80].

Abnormal expression of mitogen-activated protein kinase 7 (MAPK7) has been defined as a biomarker for tumor development in high-grade OS [81, 82]. MiR-143 has been evaluated as a tumor suppressor in many kinds of malignancies [83–85]. Down-regulation of miR-143 was found in OS tissues and cells, whereas over-expression of miR-143 can play a role in inhibiting the proliferation, migration and invasion of OS cells. Furthermore, the miRNA level of MAPK7 was reported to be negatively linked to miR-143 expression in OS tissues. Thus, MAPK7 could be a target of miR-143, and forced expressed miR-143 has been revealed to be implicated in decreasing the MAPK7 protein expression [86].

It has been revealed that miR-143 was able to suppress epidermal growth factor receptor (EGFR) via its downstream ERK/MAPK signaling cascades for negative regulation of Matrix metallopeptidase 9 (MMP-9) level in OS cell lines; therefore, miR-143, EGFR, and MMP9 have been suggested as key targets for preventing OS invasion [87]. Competitive endogenous RNAs (ceRNAs) regulatory network indicated that LINC00323, LINC00028, SNHG1 (lncRNAs), hsa-miR-7, and hsa-miR-124 are importantly implicated a new mechanism of interaction between some mRNAs (i.e., RAP1B, ATF2 and PPM1B) involved in the MAPK pathway [88].

Over-expression of hsa-miR-124 and hsa-miR-7 demonstrated to have favorable prognosis value. Decreased miR-7 level in OS has been found to be linked to poor prognosis [89]. In addition, miR-124 expression level has been revealed to be markedly lower in the metastatic OS as compared to non-metastatic OS. MiR-124 serves as a tumor suppressor by inhibiting expression of Rac family small GTPase 1 [88, 90].

### MTOR and RAF-1 Signaling MiRNAs in OS

A study demonstrated that miR-24 was decreased in OS, leading to up-regulation of lysophosphatidic acid acyltransferase β (LPAATβ) and induction of OS cell proliferation. LPAATβ has been defined to be implicated in the regulation of OS cell proliferation, partly through mTOR and Raf-1 signaling pathways [91]. Nevertheless, further clarification will need the systematic evaluation of the molecular mechanisms involved in the regulation of LPAATβ in OS.

MiR-199a-3p (1q24.3) has been suggested to be implicated in suppression of mTOR signaling via binding of the 3′UTR of mTOR. Restored miR-199a-3p expression was able to decrease mTOR and p-mTOR and enhance cell populations via increasing G_1_-phase population, leading to suppression of cellular growth, proliferation in OS cells. In another word, increased level of miR-199a-3p via transfection has been indicated to be capable of both decreasing OS cell growth and migration by enhancing G_1_-phase population, decreasing the S-phase, and restoring miR-199a-3p level [92].

Up-regulated miR-101 was capable of enhancing mTOR expression at both mRNA and protein expression level in OS, resulting in suppression of cell proliferation and promotion of apoptosis in an mTOR-dependent manner [93].

MTOR/p70S6K signal transduction pathway has been revealed to be associated with positive surgical stage and metastasis status, indicating the prognostic value of this pathway in OS patients [94].

Functional studies demonstrated that miR-99a is a key regulator of mTOR [95–97]. It has been revealed that miR-99a was negatively linked to mTOR mRNA in OS, where low miR-99a expression and high mTOR expressions were markedly linked to high surgical stage, and metastasis recurrence, therefore, miR-99a-high/mTOR-low patients showed relatively better outcomes, indicating that miR-99a-low/mTOR-high co-expression can potentially be served as a novel prognostic marker for OS [97].

### MicroRNA-15/16 cluster in OS

The miR-15/16 cluster has been considered to be involved in the suppression of tumor in many kinds of malignancies. This cluster has been indicated to target BCL2, WNT3A, RAB23 genes and other genes implicated in the G1/S transition, e.g., cyclin D1, cyclin D3, cyclin E1, and CDK6 [5, 98].

MiR-16 was demonstrated to be weakly decreased in OS, while its over-expression has been observed to be capable of suppressing IGF1R/Kras/Raf1/MEK/ERK pathway, leading to suppression of cell growth in OS, indicating that exogenous up-regulation of miR-16 may provide a therapeutic strategy in the near future [99]. In addition, restoration of miR-16 in OS cells has been indicated to be attributed to inhibition of proliferation via suppressing IGF-1R and the Ras/Raf/MAPK pathway, while MAPK activation was capable of inducing proliferation and anti-apoptotic pathways in OS cells [5, 100].

MiR-16-1-3p and miR-16-2-3p together with miR-16-5p were demonstrated to be down-regulated in OS and mouse model with engineered WWOX gene [101–104], while tumor suppressive effects of 16-1-3p and miR-16-2-3p were markedly higher in OS than that of miR-16-5p strand [105]. On the other hand, obligatory knock-outs of neither miR-16-1 nor miR-16-2 could contribute to OS in mice model [106, 107]. These findings need rigorous analysis in the light of some interpretation including additional oncogenic events for development OS in mice or involvement of reduction of these miRNAs in later stages of tumorigenesis in mice or implication of both events, and/or the possibility of differences mechanisms for both mice and humans [105]. High expression levels of miR-16-1-3p and miR-16-2-3p together with miR-16-5p have been found to be associated with a decrease in Akt Ser473 phosphorylation that is compatible with over-expression of PI3K/Akt pathway in osteoblasts with FGFR2 up-regulation [105, 108]. These miRNAs exhibited anti-survival and pro-apoptotic activities, as well as anti-invasive and chemoresistance-lowering effects in human OS cells at endogenous expression.These mimics have been suggested as key targets for improving the outcomes of chemotherapy in OS [105].

### P53-linked miRNAs in OS

Based on the data presented in the literature, aberrant expressions of miR-34a, miR-34b and miR-34c have been markedly linked to p53 status, indicating the possibility of the regulation of the Mir-34 gene by p53. The inactivating mutations of p53 have been revealed to be often associated with the reduction of miR-34a in tumors [109, 110].

MiR-34a, a member of miR-34 s family, is a transcriptional target of p53 tumor suppressor, which is capable of suppressing cell proliferation and metastasis in OS by reducing the cMet gene [111]. miR-34a plays a substantial role in inhibiting tumorigenesis via down-regulation of its targets, e.g., Cyclin D1, E2F3, E2F5, CDK4, CDK6, N-myc, c-Met and Bcl-2 [112–114]. On the other hand, the p53 network has been indicated to be able to inhibit tumorigenesis via activation of its transcriptional targets. MiR-34 may play a key role in suppressing inappropriate cell proliferation and over-expression of miR-34a is capable of decreasing c-Met protein and miRNA, resulting in inhibition of the tumor growth and metastasis in OS although other putative miR-34a target genes may be potentially involved in the progression of OS. Taken together, the absence of miR-34a has been found to attribute to the development of a variety of malignancies [115, 116].

MiR-34a has been reported to be capable of regulating genes involving in DNA damage and repair. MiR-34a was found to decrease in OS, and its expression has been suggested to be associated with the expression of its target genes (i.e., CDK6, E2F3, Cyclin E2,and Bcl-2) partly in a p53-dependent manner, and subsequently resulted in the miR-34 s-induced cell cycle arrest, and apoptosis [117]. On the other hand, it has been indicated that miR-34a is implicated in suppression of OS growth by reduction of Eag1 expression [118]. The p53-dependent miR-34c decreased runt-related transcription factor 2 (RUNX2) in OS, and Nutlin-3-mediated stabilization of p53 was found to be capable of promoting miR-34c level and reducing RUNX2, resulting in inhibition of U2OS cell proliferation [119]. MiR-34a and miR-199a-3p have been demonstrated to have important roles in blocking cell growth and elevating cell apoptosis via p53 signaling pathway by down-regulating its targets (mTOR, MET and MDM4 [an inhibitory factor of TP53] in OS [120]. An investigation reported that p53-associated miR-34a and miR-192 expression levels can be served as a prognostic marker for risk stratification in OS [121].

MiR-215 has been found to be linked to cell cycle control, cell proliferation [122], and play a key role in p53-mediated chemoresistance, where miR-215 over-expression has been found to be attributed to resistance to methotrexate (MTX) and tomudex (TDX) in OS cell lines [123]. A growing body of evidence suggests that miR-34a, miR-192, and miR-215 can be considered as prognostic markers candidate in OS.

### Notch signaling-related micro RNAs

Increasing evidence indicates that miR-199b-5p expression was markedly increased in OS tissues, when compared with normal tissues. Furthermore, the miR-199b-5p inhibitor was found to be capable of altering expression levels of Notch pathway components including JAG1, Notch1, HES1, Dll1, Dtx1 via regulation of HES1 and Dtx1 expression levels [124]. As demonstrated previously, the balance between HES1 and Dtx1 is implicated in regulating Notch signaling [124, 125]; therefore, miR-199b-5p play a key role in the regulation of Notch signaling in OS.

A study indicated that over-expression of miR-199b-5p was linked to adverse outcomes including high tumor grade, metastasis, recurrence, and shorter overall survival in patients suffering from OS [126]. Notch signaling is involved in the development of many kinds of cells and tissues (e.g., bone development) via affecting stem cell renewal, proliferation, differentiation, etc. As a matter of fact, this pathway plays a key role in keeping the balance between proliferation and differentiation and its changes (i.e., increased expression of Notch ligand and receptors) can lead to the development of cancers such as OS [127–130].

Accumulating evidence indicates tumor suppressor miR-34a is capable of regulating p53 and Notch signaling in OS [131, 132]. Notch signaling components have been demonstrated to be increased in primary OS [133], and the miR-34 reduction was revealed to be associated with the genetic and epigenetic changes of miR-34 genes in primary OS [117, 134].

Furthermore, miR-34a and miR-200b were found to play a crucial role in the regulation of a number of genes including Notch-1, VEGF, MMP-2 and MMP-9 in OS cells. Diallyl trisulfide (DATS), an organic trisulfide derived from Allium vegetables, is capable of suppressing development and aggressiveness of OS through down-regulating its downstream genes (MMP-2, MMP-9 Hes-1, and VEGF) and increasing a panel of tumor-suppressive microRNAs, e.g., miR-34a, miR-200b/c, miR-143, and miR-145, which are usually lost in OS; thus they are considered as new targets for developing therapeutic strategies [135].

In addition, re-expression of both miR-34a and miR-200b via transfection were related to down-regulation of Notch-1 expression, resulting in suppression of cell proliferation, invasion and angiogenesis in OS. Additionally, miR-200b and miR-200c have been reported to be decresed in OS cells, and Notch-1 inactivation was found to be implicated in up-regulation of miR-200b and miR-200c. Additionally, DATS was capable of inducing re-expression of miR-200b and miR-200c, indicating a valuable component for reverting aggressiveness of disease [135].

MiR-34c is implicated in suppressing osteoblast differentiation and enhancing osteoclastogenesis partially by inhibiting Notch signaling components e.g., Notch1, Notch2, and Jag1 in mice. Further development is needed in-depth understanding of miR-34 and Notch pathway interactions that underlie their regulation to provide therapeutic strategies modulating miR-34 signaling [134].

### MiRNAs involved in apoptosis

Apoptosis is considered as a homeostatic mechanism which can be triggered by two major apoptotic pathways including mitochondria-mediated intrinsic pathway and death receptor-mediated pathway (extrinsic apoptotic pathways), by which is capable of activating a group of cysteine proteases including caspase-9 and- 8, respectively. These caspases play an important role in the activation of caspase-3, -6, and -7, which are capable of promoting cleavage of different cellular proteins in order to induce cell death [136, 137].

MiRNAs are not only responsible for regulation of the apoptotic extrinsic apoptotic pathways via different key junctions such as TRAIL-R, Fas ligand (FasL), TWEAK and IP3R, BIRC5, and CASP7, but also play an important role in the regulation of intrinsic pathway via junctions such as cathepsin and Bcl-2 family members and inflammation through IL3RB and PI3K [137].

### Intrinsic apoptotic pathway involved MiRNAs in OS

Down-regulation of miR-133a was found in primary OS to be linked to tumor progression and prognosis of disease [138]. It should be taken into consideration that molecular mechanisms by which miR-133a play its role in cell proliferation, and invasion in OS will need further development. A study indicated miR-133a is involved in inhibition of progression and metastasis via targeting insulin-like growth factor 1 receptor (IGF-1R) in OS and indirectly suppresses the AKT/ERK signaling pathways [139]. IGF-1R is participated in regulation of cell proliferation, and apoptosis [140]. Therefore, miR-133a might be a target and effective biomarker for metastasis and prognosis of OS. MiR-133b expression level has been recorded to be decreased in OS, and its over-expression was found to reduce BCL2L2, MCL-1, IGF1R, MET and FAK and inhibit Akt activation, resulting in inhibition of cells proliferation, migration, and invasion, thus leading to the promotion of apoptosis in OS cells [141]. Both BCL2L2 and MCL-1 are defined to be as members of the Bcl-2 family, which are capable of increasing cells survival and therefore exhibit anti-apoptotic activity via the mitochondrial signaling pathway [142, 143].

It has been indicated that loss of the miRNA29a level may be involved in up-regulation of BCL2 and MCL1, leading to resistance of cells to apoptosis, and progression of OS, while over-expression of miRNA29a was associated with increased E2F1 and E2F3 expression levels as a tumor suppressor and loss of both BCL2 and MCL1 expression levels [144]. The E2F is a panel of genes that have a key role in the regulation of the cell cycle and DNA synthesis in mammalian cells [145].It has been revealed that knockdown of miR-29 result in suppression of cell proliferation and induction of apoptosis in OS by inducing PUMA through inhibition of TGF-β1 levels, suggesting miR-29 anti-tumor activities [146].

### Extrinsic apoptotic pathway involved miRNAs in OS

Over-expression of miRNA cluster 17-92 and its two paralogs (i.e., 106a-363 and 106b-25) are indicated to be an oncogenic event in OS cell lines. Accumulating evidence suggests that expression of miR-17, miR-18a, miR-92a, and miR-106b have been contributed to FAS repression [147].

Furthermore, over-expression of the miR-17-92 cluster, particularly miR-20a level was demonstrated to be involved in FAS suppression in OS cell lines that contributes to tumor cell survival and metastasis (lung metastases) in OS cells [148]. The involvement of Fas-FasL signaling in tumor progression and suppression can be controversially different in many kinds of tumors. It not only plays an important role in apoptosis as a signal, but also in some examples stimulates cell proliferation via nonapoptotic signaling [147, 149, 150].

Suppression of BIM as a pro-apoptotic gene was induced by the miR-17-92 cluster in many types of tumors, and osteoblasts [151]. However, only the miR-17 expression level has been found to contribute to decreased pro-apoptotic BH3-only gene (BIM) expression in OS [147]. Current evidence suggests a superordinate role of the miR-17-92 cluster in OS biology, where several pathways and mechanisms may be involved in the development of OS [147, 152].

### Invasion-metastasis-related microRNAs in OS

As indicated, miR-17–92 cluster (i.e., miR-17, miR-18a, miR-19a/b, miR-20a, and miR-92), especially miR-20a is involved in development of OS and metastasis formation [148].

Increased level of MiR-93 seems too contributed to OS progression and invasion [153]. Over-expression of miR-93, miR-181c, and miR-27a, have been previously reported in OS [153, 154]. MiR-23a–27a–24-2 cluster has been found to be involved in retaining the osteocyte phenotype and progression [155]. Increased miR-27a was capable of promoting invasion, and proliferation in metastatic sites, leading to the enhancement of osteoblast differentiation. Additionally, targeting of peroxisome proliferator-activated receptor gamma (PPARγ) through miR-27 has been considered to be a second function for maintaining osteoblast phenotype during differentiation process [155].

Decreased expression of miR-183 was reported to be associated with lung metastases and local recurrence of OS. In addition, tumor suppressive role of MiR-183 was found to be implicated in the inhibition of Ezrin expression and suppression of MAPK/ERK activation, therefore, miR-183-Ezrin-MAPK/ERK axis was suggested to prevent progression and metastasis in OS [156]. Accordingly, a study indicated that miR-183 was capable of suppressing cell migration and invasion and metastasis thought down-regulation of the Ezrin expression [157].

Another study revealed that dysregulation of miR-182 and miR-183 may play a crucial role in the development of OS [158]. The small molecule inhibitors NSC305787 and NSC668394 have been capable of inhibiting Ezrin and preventing OS metastasis. Additionally, ezrin silencing was suggested to modulate the expression of PI-PLC in the human OS, and consequently can serve as the basis for the prevention of OS progression [159, 160]. Decreased expression level of miR-183 has been demonstrated to be negatively linked to Ezrin mRNA over-expression in OS, thus this event was found to be associated with clinicopathological characteristics including advanced grade, metastasis, recurrence, chemoresistance, and poor overall survival, suggesting that it might be a novel potential biomarker for predicting prognosis and aggressiveness of OS [161].

Decreased expression of miR-143 has been demonstrated to be linked to the lung metastasis of OS cells via enhancing invasion by matrix metalloproteases-13 over-expression as a downstream mediator of miR-143, indicating that it might be a novel target for OS metastasis.

Down-regulation of ROCK1-related miRNAs (i.e., miR-129-5p, miR-198, miR-144, and miR-145, miR-150, miR-202-5p, miR-340, miR-335) has been reported to be linked to OS progression and metastasis via targeting ROCK1 [162–169]. Thus, OCK1 can be suggested as a novel therapeutic target in patients suffering from OS.

A study also demonstrated that over-expression of miR-20b contribute to the suppression of the invasion and growth of OS cells, and inhibition of the HIF-1α and VEGF pathway proteins, whereas the suppression of miR-20b was capable of showing the reverse findings. In addition, miR-20b showed inhibition of the tumor cell process by suppressing HIF-1α level [170].

A deep RNA sequencing indicated that miR-612, miR-1197, miR-193b-3p, miR-1262, miR-144-3p, and miR-1269a may contribute to OS metastasis, where further development needs an in-depth understanding of mechanisms and targets involved in OS metastasis via regulation of these miRNAs [171].

Activator protein-1 transcription factor, (c-FOS) has been suggested as an oncogene in OS and its up-regulation was found to be able to induce OS formation via cooperating with c-jun in transgenic mice [172, 173]. Up-regulation of both c-myc and c-FOS have been reported in relapsed OS, which could contribute to the development of OS and metastasis [172],therefore, it can be concluded that the synchronous increase of c-myc and c-FOS might be a novel potential predictor for metastasis in primary OS.

Increased expression of c-FOS has been found to reverse the suppressive role of miR-101 over-expression on proliferation and invasion of OS cells by targeting of c-FOS. Therefore, c-FOS can be served as novel therapeutic targets for OS.

## 14Q32-associated miRNAs cluster in OS

Based on the evidence presented in the literature, decreases in the network of 14q32 miRNAs (miR-382, miR-369-3p, miR-544, and miR-134) is capable of both mediating the regulation of cMYC transcript by increasing cMYC protein and elevating the level of miR-17-92 clusters [174]. Up-regulation of miR-17-92 has been attributed to the aberrant cell division and evading apoptosis [175], while lowering the level of cMYC is linked to apoptosis in OS cell. 14q32 miRNAs have been reported to be involved in suppression of tumor development and their expressions may be negatively associated with the mitotic potential of osteoblasts. In addition, deregulation of 14q32 miRNA cluster may play a key role in osteosarcoma genesis [176].

An increasing body of evidence suggests that 14q32 miRNA-cMYC-miR-17–92 miRNA network can be involved in the pathogenesis of OS [174]. Another study indicated that single nucleotide polymorphism (SNP) at the 14q32 miRNA cluster (rs12894467, rs58834075, rs12879262, and rs61992671) could contribute to the OS susceptibility in the Spanish population [176, 177].

Further development will need new large-scale studies, functional analyzses and in-depth understanding of the molecular mechanisms that underlie regulation and mutation of the 14q32 miRNA cluster in OS.

### Bioinformatics Analysis of MiRNAs in OS

In this review, OS-associated miRNAs including oncomiRNAs and circulating tumor suppressor miRNAs were retrieved from the literature. Then, experimentally validated miRNA-target was obtained from the miRTarBase web server in at least two experimental methods. Moreover, the interaction network of all OS-associated miRNAs and their targets was reconstructed using Cytoscape v.3.6.1.

**Fig. 2:**
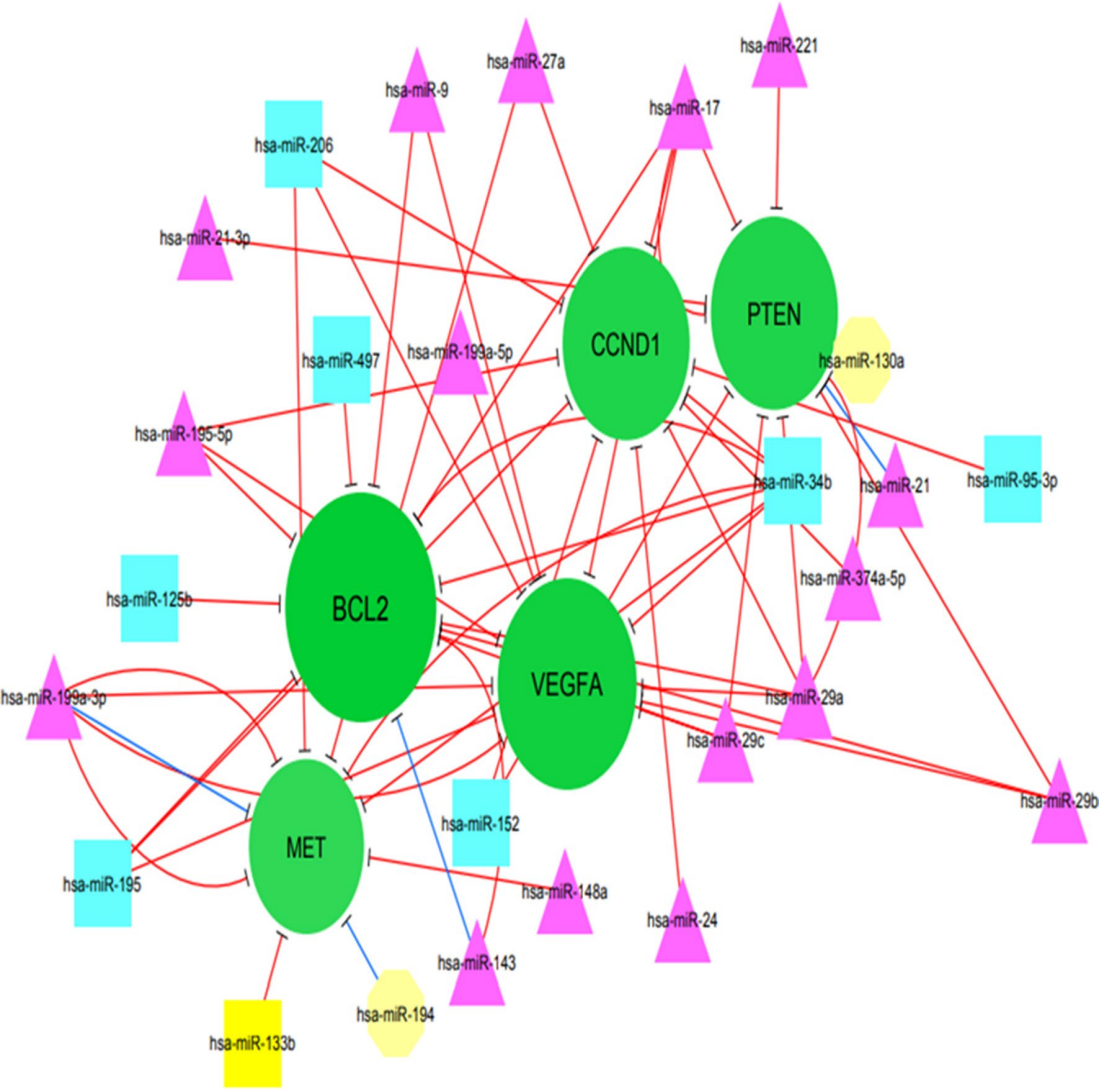
the sub-network showed the potential hub gene nodes (nodes with over 9 in degrees): BCL2 (13), VEGFA (12), CCND1 (11), PTEN (11), MET (10) and miRNAs that regulated them

In addition, the topology of the network was analyzed based on degree metrics in order to find the most important nodes. According to the findings, 5 nodes with large degrees in our large network (i.e., > 9; green nodes) including B cell lymphoma 2 protein (BCL2), vascular endothelial growth factor A (VEGFA), CCND1 (Cyclin D1), phosphatase and tensin homolog (PTEN) and MET were identified as potential hub nodes, (Additional file 1 and Fig. 2).

#### BCL2

BCL2 as key antiapoptosis regulator causes not only inhibition of cell apoptosis via removal of pro-apoptotic genes, but also could play a role in increasing anti-apoptotic genes and cell viability [178]. It is noteworthy that the weak expression of miR-449a in OS was linked to high tumor stage and poor prognosis. MiR-449a is capable of inducing apoptosis via inhibition of BCL2 expression [179]. Furthermore, reduction of miR-143 expression was observed in OS, and its inhibitory role in the development of OS was regulated through the inhibiting Bcl-2, leading to induction of apoptosis [180]. MiR-34a has been reported as an important mechanism of lead-inhibited tumor invasion and metastasis in OS and its inhibitory role may be partially associated with decreased C-IAP2 and Bcl-2 expression [181]. BCL-2 and its family members have been observed to be involved in many types of cancer. Therefore, it might be an important target for the development of the therapeutic approach in the future.

#### VEGFA

VEGFA gene is defined to play a crucial role in cellular proliferation, survival, and angiogenesis in OS [5, 182]. VEGFA was introduced as a poor prognostic marker for tumor-free survival in OS, suggesting its potential for anti-VEGF therapy [183]. VEGFA expression was found to be increased in the OS cell line (SAOS-2) and tissues. It has been also revealed that down-regulation of miR-497 could promote OS cells growth and cisplatin resistance by PI3K/Akt signaling via direct targeting VEGFA, while its over-expression was linked to a reverse event. MiR-497 has been reported to be capable of modulating proliferation and apoptosis via targeting VEGFA/PI3KAKT pathway in OS [184]. MiR-134 has been found to be dramatically involved in the inhibition of AKT activation and proliferation of cell nuclear antigen expression in OS. As a result, miR-134 was introduced as a tumor suppressor via attenuating VEGFA/VEGFR1 signaling to decrease OS progression and angiogenesis [185]. Ample evidence indicates that VEGFA/VEGFR1 signaling may be a therapeutic target for many kinds of cancer, such as OS when previous studies revealed its prognostic role on OS [186, 187].

#### CCND1

CCND1 participates in the regulation of cell cycle progression [188]. Accumulating evidence suggests that CCND1 was over-expressed in human cancers such as OS that exerts an oncogenic role in the progression of OS via regulating cell proliferation, the cell growth, migration, invasion, and metastasis in vitro and in vivo [189–191]. In addition, over-expression of CCND1 was found to be linked to shorter overall survival in OS patients [190]. A study indicated that miR-195 expression could act as a tumor metastasis suppressor via attenuating CCND1 [190]. MiR-466 is not only responsible for inhibition of OS proliferation and cell cycle, but also play an important role in promoting apoptosis, leading to inhibition of OS progression via targeting CCND1 [192]. Regarding the importance of CCND1, targeting of CCND1 may be useful to develop a therapeutic target for preventing the rapid progression and metastasis in individuals suffering from OS.

#### PTEN

PTEN is a tumor suppressor gene that plays a key role in tumor cell growth, migration, and invasion, as well as apoptosis and serves as a key regulator in many types of cancer [193, 194]. It has been indicated that miRNA-21, PI3K, and AKT are highly expressed in the OS cell line. Over-expression of miRNA-21 not only promotes proliferation, but is also linked to overexpression of PI3K/AKT signaling pathway proteins via attenuating the expression of PTEN, suggesting that PTEN might serve as a target of miR‐21 [195]. Additionally, miRNA-21 knockdown was capable of suppressing OS cell proliferation by promoting PTEN and TGF-β1 pathway [79, 196]. In OS cells, miRNA-300 could be importantly involved in the regulation of the Ubiquitination of PTEN via the CRL4BDCAF13 E3 ligase [197].The miR-524 expression is involved, not only in increased cell proliferation, but also in the attenuating apoptosis via activation of the PI3K/AKT signaling pathway by suppressing PTEN [198]. Based on the data presented herein, PTEN can be considered as a therapeutic target for OS.

#### Met

Different receptor tyrosine kinases (RTKs) and their ligands were highly expressed in OS, e.g., c-Met or tyrosine-protein kinase, EGFR, PDGFR, VEGFR, ErbB2, IGF-1R, NGFR [199–203]. A study reported that miR-1 and miR-133b were weakly expressed in OS cell lines, that their ectopic expression could cause suppression of cell proliferation and invasiveness via attenuating Met expression, thus supporting the important role of miR-1 and miR-133b in OS via Met modulation [204]. Furthermore, miR-133b was observed to be capable of attenuating the expression level of its targets (i.e., Met, BCL2L2, IGF1R, and MCL-1) and may serve as a tumor suppressor in OS [141]. MiR-199a-3p has been demonstrated to attenuate some oncogenes and antiapoptotic genes (i.e., MET, mTOR, MCL-1 and Bcl-X_L_, Stat3), suggesting its potential tumor suppressive role [205].

Additional file 1 and Fig. 2 depict eclipse (circle); target mRNA; octagon node; triangle.

node; rectangle node; miRNAs). In addition, green nodes show miRNA target genes that were mapped based on the size, color density, and label font size (Minimum:1 Maximum:13). Additionally, edge color is indicative of the source of data as blue and red colors that depicts literature data and miRTarBase, respectively. Moreover, the cluster analysis of the network was performed using ClusterOne plugin that resulted in a significant cluster with three nodes (p-value: 0.0296732) including hsa-miR-124, SPHK1, and B7-H3.

### Strategies for microRNA-based therapy in OS

Ample evidence has also attributed an important role to miRNAs in the development of OS by regulating proliferation, metastasis, invasion, apoptosis, and angiogenesis. Based on the available data, aberrant expression levels of miRNAs have been documented in OS. Several miRNAs have been confirmed as cancer biomarkers by the US Federal Drug Administration (FDA) in clinical trials. A large number of studies are underway to assess circulating miRNAs for providing novel diagnostic and prognostic markers, as well as therapeutic targets that will be valuable and non-invasive for patients suffering from cancer [206–208].

Regarding the field of liquid biopsy, there are still technical challenges for detecting circulating miRNAs including sample handling, and isolation and normalization of miRNAs, i.e., techniques of exosome isolation and purification of RNA, normalization of an exogenous reference RNA for decreasing variation associated with RNA degradation [208].

**Fig. 3:**
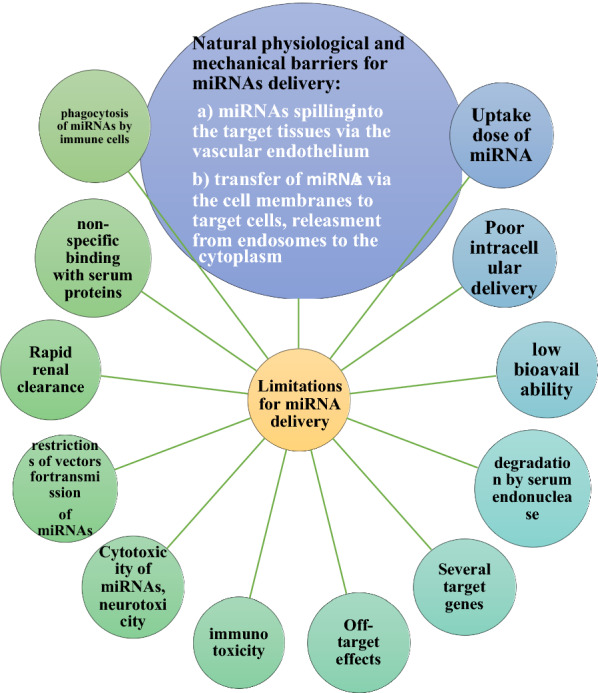
Limitations and challenges for miRNA delivery into cancer in clinical trials

Further development will need an in-depth understanding of the target molecules of miRNAs and molecular interference in OS that underlie its influence in order to develop therapeutic strategies because miRNAs replacement therapy can be a valuable strategy in cancer treatment and draw interest from studies. To the best of our knowledge, all registered clinical trials at clinicaltrials.gov are approximately based on the detection of miRNAs expression in many kinds of disease such as cancer [209, 210], where there were limitations and challenges for miRNA delivery into cancer in clinical trials (Fig. 3). Currently, two miRNA‐based strategies including miRNA mimics (e.g., restoration, replacement, or over-expression of miRNAs) and antagomiRs (e.g., miRNA down-regulation and inhibition) are considered in regard to functions of miRNAs for developing miRNA-based therapy [210, 211].

### MicroRNA mimics and antagonists

MiRNA mimics are applied as an innovative approach for restoring the activity of tumor suppressive miRNAs via replacing down-regulated miRNA by applying chemically designed (2′-O′methoxy) double-stranded RNA-like molecules. MiRNA mimics can be used via loading into RNA-induced silencing complex (RISC), which is specifically capable of downstream inhibiting of the target mRNAs. It is worth noting that miRNA mimics not only directly restore loss of tumor suppressor miRNA in cancer cells, but also can shed light on the therapeutic approach with normal expression levels of miRNA [212, 213]. MiRNA mimics as a new avenue will be greatly beneficial by the administration of miRNA-mimetic agents that their potential therapeutic effects can be truly evaluated for cancer management in clinical trials. However, challenges in the field of miRNA-mimetic agent delivery remind, where various approaches have recently been investigated for delivery agents and their delivery will be greatly favorable in achieving affordable, safe, and efficient delivery.

There is interest in developing miRNA-targeting therapies, where miRNA inhibitors and oligomers are developed for inhibiting miRNA biogenesis, including anti‐miRNA oligonucleotides (AMO), locked‐nucleic‐acid antisense oligonucleotides (LNAs), miRNA masks, antagomirs, miRNA sponges, nanoparticles (NPs), multi-target anti-microRNA antisense oligonucleotide (MTg-AMOs) [214–217].

AMOs (i.e., single-stranded, DNA-like molecules with chemical modification, antisense oligonucleotides (ASOs) have been developed not only for inhibiting miRNA function to specific signaling pathways, but also for blocking the function of malignancy-related miRNA. They are capable of providing antisense oligonucleotides (ASOs)–miRNA duplex by watson–crick binding, resulting in RNase H-mediated miRNA gene cleavage.

MiRNA antagonists-AMO along with complementary sequences are developed to be complementary to a targeted miRNA gene, where are specifically able to block miRNA function and RISC assembly, leading to over-expression of tumor suppressor genes [213, 218]. It is noteworthy that targeted miRNA can be considered as a master predictor of response to miRNA antagonist therapy for certain tumors [213]. Providing chemically modified miRNA mimics with an oligonucleotide render a considerable challenge and the different proprietary modification approaches are suggested in chemically modified off-the-shelf miRNA mimics [219].

Nevertheless, the configuration of miRNAs is impossible to be processed using RISC [220]. Regarding the miRNA‐reduction therapy, a single miRNA is not appropriately sufficient for clinical therapy because of the presence of different oncomirs and targets. Another challenge of miRNA‐reduction therapy is described to be the degradation of oligonucleotides via endonucleases in the blood [217, 221]. Current evidence suggests an important role for importing exogenous miRNAs in miRNA replacement therapy via inhibition of proliferation or induction of apoptosis in tumor cells [217, 222].

LNAs are defined as modified RNA nucleotides, that belong to a class of antisense ON’s; current evidence has also attributed a role to the methylene bridge in LNAs in conformational locking of the ribosome and shows remarkable binding affinity to a single-stranded RNA [223]. LNAs have attracted the interest of researchers for developing therapeutic strategies in cancer. These molecules are capable of increasing target affinity and are stabilized to elevate stability against nuclease degradation. Suppression of oncomirs has been described as a strategy using LNAs, synthetic anti-miRNAs, antagonists, and TS miRNAs, as well as ASOs [224, 225].

Another approach is described to block the function of a miRNA of interest by applying a miRNA sponge. The miRNA sponge with several complementary 3′ UTR mRNA sites for targeted miRNA is capable of inducing continuous loss-of-function phenotypes for targeted miRNA in cell culture and transgenic organisms, as well as virally infected cells [211, 226]. Many miRNA sponge types have been previously constructed as target mimics such as miRT sequences decoys, and lentivirus-mediated antagomir (miRNA erasers) [227–230].

MiRNA sponges can be potentially applied for targeting the family of miRNAs, as compared to antisense oligonucleotides which are able to target a single miRNA [226]. However, there are some challenges herein; it should be taken into consideration that the use of sponge methods can show different levels of inhibition in many contexts. Furthermore, there are less free miRNAs, when cells exhibit a large pool of endogenous targets for targeted miRNA family; therefore, it can be concluded that a lower dose of sponge RNA can be sufficiently effective for blocking a miRNA of interest. Nevertheless, determining the effectiveness of sponge (efficacy) is currently considered to be challenging as compared to the validation of successful miRNA deletion. The validation of efficacy for sponge method is available using methods such as cell culture [231]. On the other hand, therapeutic applications of miRNAs need a favorable invivo delivery device or mechanism [232], where delivery is considered as one of the major challenges.

## Conclusion

Currently, ample evidence suggests that miRNAs are key regulators of tumor initiation, development, dissemination and the inhibition of proliferation or induction of apoptosis in tumor cells, in parallel, targeting of miRNAs should be also being considered for therapeutic strategies, which are related to suppression of tumor growth and metastasis in OS.

Our bioinformatics analysis revealed that BCL2 (antiapoptosis regulator), VEGFA (cellular proliferation, survival, and angiogenesis), CCND (oncogenic properties: cell proliferation, the cell growth, migration, invasion, and metastasis), PTEN (tumor suppressor gene) and MET (oncogenic properties) were potential hub gene nodes in the subnetwork of OS. MiR-449a and MiR-34a are considered to be involved in the apoptosis pathway and suppression of OS, respectively, via regulating BCL2.

Overexpression of miR-497 and miR-134 may be involved in suppression of OS progression via targeting VEGFA and VEGFA/VEGFR1, respectively. In addition, miR-195 and miR-466 expressions could act as a tumor metastasis suppressor and OS progression inhibitor, respectively, via targeting oncogenic CCND1. Furthermore, miR-524 and miRNA-21 knockdown may be capable of inhibiting OS cell proliferation by promoting PTEN. Additionally, miR-1, miR-133b and MiR-199a-3p may be considered key tumor suppressors in OS through inhibition of cell proliferation and invasiveness via attenuating Met expression.

Further studies are required to fully understand the biological functions of miRNAs derived from serum/plasma, tissues, and other biological fluids, as valuable regulators and promising markers are implicated in the development of OS. Although there are lots of challenges for normalization of miRNA, miRNAs may be valuable biomarkers for determining risk of OS progression. Underlying functions and biological mechanisms of various miRNAs are currently far from favorably understood. At present, there are two key miRNA‐based strategies including miRNA mimics and antagomiRs to develop miRNA-based therapy for modulating tumor microenvironment and inhibiting tumor development.

## **Supplementary information**

**Additional file 1.** Network analysis.

## Data Availability

Not applicable.

## Abbreviations

MiRNAs: MicroRNAs
OS: Osteosarcoma
ECmiRNAs: Extracellular miRNAs
NGS: Next-generation sequencing
HDL: High density lipoprotein
AGO: Argonaute
MET: Mesenchymal epithelial transition
PDGF: Platelet-derived growth factor
PTEN: Phosphatase and tensin homolog
LOH: Heterozygosity
MAPK7: Mitogen-activated protein kinase 7
EGFR: Epidermal growth factor receptor
MMP-9: Matrix metallopeptidase 9
LPAATβ: Lysophosphatidic acid acyltransferase β
RUNX2: Runt-related transcription factor 2
MTX: Methotrexate
TDX: Tomudex
DATS: Diallyl trisulfide
IGF-1R: Insulin-like growth factor 1 receptor
PPARγ: Peroxisome proliferator-activated receptor gamma
SNP: Single nucleotide polymorphism
VEGFA: Vascular endothelial growth factor A
PTEN: Phosphatase and tensin homolog
RTKs: Receptor tyrosine kinases
FDA: Federal Drug Administration
RISC: RNA-induced silencing complex
AMO: Anti-miRNA oligonucleotides
LNAs: Locked-nucleic-acid antisense oligonucleotides
NPs: Nanoparticles
MTg-AMOs: Multi-target anti-microRNA antisense oligonucleotide
ASOs: Antisense oligonucleotides
