# Supplementary figures and images for: MicroRNAs signatures, bioinformatics analysis of miRNAs, miRNA mimics and antagonists, and miRNA therapeutics in osteosarcoma
Source: Cancer Cell Int. 2020 Jun 17;20:254. doi: 10.1186/s12935-020-01342-4 (PMC7302353; doi:10.1186/s12935-020-01342-4)

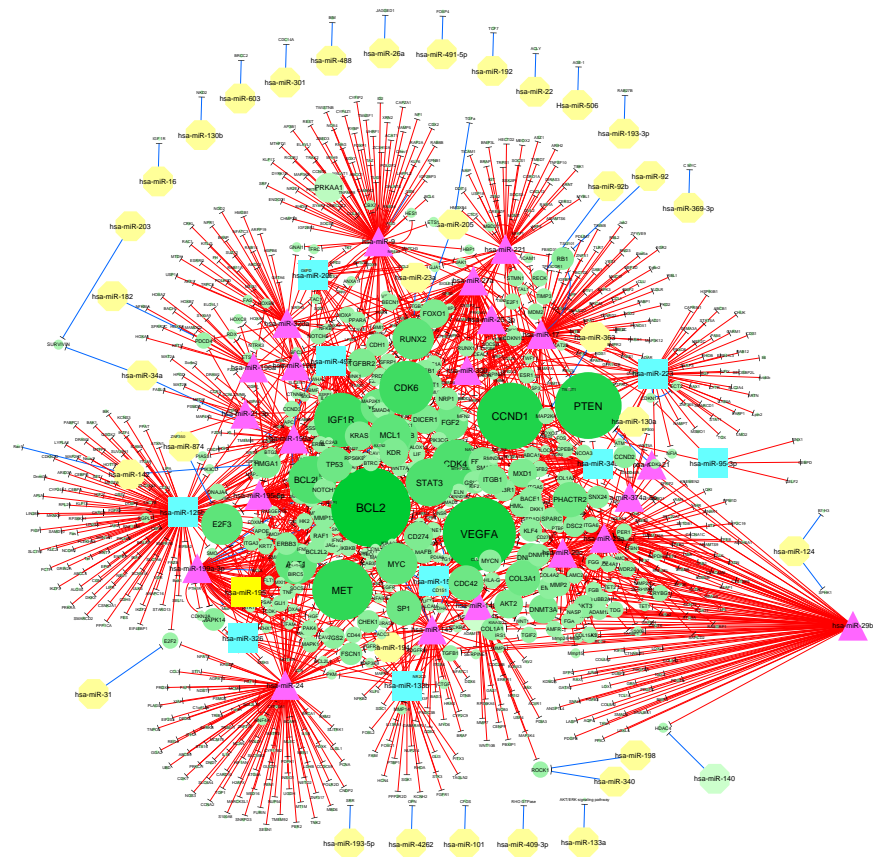

Supplement: Supplementary file 1 — Additional file 1. Network analysis. [file 12935_2020_1342_MOESM1_ESM.pdf]
